# High rate and large intercentre variability in retreatment of retinopathy of prematurity in infants born <24 gestational weeks

**DOI:** 10.1136/bmjophth-2020-000695

**Published:** 2021-04-21

**Authors:** Pia Lundgren, Lena Jacobson, Anna-Lena Hård, Abbas Al-Hawasi, Eva Larsson, Lotta Gränse, Marie Saric, Birgitta Sunnqvist, Kristina Tornqvist, Agneta Wallin, Gerd E Holmstrom, Lois LE Smith, Eva Morsing, Ann Hellström

**Affiliations:** 1 The Sahlgrenska Centre for Pediatric Ophthalmology Research, Department of Clinical Neuroscience, Institute of Neuroscience and Physiology, Sahlgrenska Academy, University of Gothenburg, Gothenburg, Sweden; 2 School of Medical Sciences, Faculty of Medicine and Health, Örebro University, Örebro, Sweden; 3 Department of Clinical Neuroscience, Karolinska Institutet, Stockholm, Sweden; 4 Department of Clinical and Experimental Medicine, Linköping University, Linköping, Sweden; 5 Institution of Neuroscience/Ophthalmology, Uppsala University, Uppsala, Sweden; 6 Department of Clinical Sciences, Ophthalmology, Skåne University Hospital, Lund University, Lund, Sweden; 7 Department of Clinical Sciences, Ophthalmology, Umeå University, Umeå, Sweden; 8 Department of Ophthalmology, Länssjukhuset Ryhov, Jonkoping, Sweden; 9 St Erik Eye Hospital, Stockholm, Sweden; 10 Department of Ophthalmology, Boston Children's Hospital, Harvard Medical School, Boston, Massachusetts, USA; 11 Department of Pediatrics, Clinical Sciences Lund, Skåne University Hospital Lund, Lund, Sweden

**Keywords:** retina

## Abstract

**Objective:**

Prematurity is a major risk factor for retinopathy of prematurity (ROP). We aimed to elucidate ROP prevalence, treatment and retreatment in infants born before 24 gestational age (GA) weeks in a Swedish cohort.

**Methods and analysis:**

Infants with completed ROP screening, born at <24 GA weeks, 2007–2018 in Sweden were included. Data of GA, birth weight (BW), sex, neonatal morbidities, maximal ROP stage, aggressive posterior ROP (APROP), ROP treatments, treatment modality and treatment centre were retrieved.

**Results:**

In total, 399 infants, with a mean GA of 23.2 weeks (range 21.9–23.9) and a mean BW of 567 g (range 340–874), were included. ROP was detected in 365 (91.5%) infants, 173 (43.4%) were treated for ROP and 68 of 173 (39.3%) were treated more than once. As the first treatment, 142 (82.0%) received laser and 29 (16.1%) received intravitreal injection of antivascular endothelial growth factor (anti-VEGF). Retreatment was performed after first laser in 46 of 142 (32.4%) and in 20 of 29 (69.0%) after first anti-VEGF treatment. Retreatment rate was not associated with GA, BW or sex but with APROP, treatment method (anti-VEGF) and treatment centre where the laser was performed (p<0.001). Twenty eyes progressed to retinal detachment, and two infants developed unilateral endophthalmitis after anti-VEGF treatment.

**Conclusion:**

Infants, born at <24 weeks’ GA, had high rates of treatment-warranting ROP and retreatments. Treatment centre highly influenced the retreatment rate after laser indicating that laser treatment could be improved in some settings.

**Key messages**

**What is already known about this subject?**

- Immaturity per se is a major risk factor for developing sight-threatening retinopathy of prematurity.

**What are the new findings?**

- The most immature infants, born before 24 weeks’ gestational age, are at high risk of severe retinopathy of prematurity requiring multiple treatments. Rates of retreatments and sight-threatening complications are high, and retreatment rates after laser therapy vary between treatment centres.

**How might these results change the focus of research or clinical practice?**

- The variation in retreatment rate after laser therapy between centres indicates that laser treatment may be improved in some centres reducing the number of episodes of general anaesthesia and possibly reducing sight-threatening complications.

## Introduction

Medical advances have greatly increased the survival of infants born at the limit of viability during the last decades, but their neurodevelopmental long-term outcomes are still a matter of concern.^1–3^ Immaturity per se is the main risk factor for sight-threatening retinopathy of prematurity (ROP).^4–6^ Treatment failure in aggressive ROP or incomplete screening may result in severe visual impairment.^7 8^ Treatment of ROP suppresses retinal vascular endothelial growth factor (VEGF), which is overexpressed in proliferative ROP which, in the worst case, progresses to retinal detachment.^9^ Laser therapy has been the treatment of choice for severe ROP in the last decades.^10^ However, intravitreal injections with antivascular endothelial growth factor (anti-VEGF) drugs are becoming more frequently used especially in infants with aggressive posterior ROP (APROP), which carries a high risk for recurrence and unfavourable outcomes after treatment.^11^

Previous studies evaluating ROP outcomes include a limited number of infants with gestational age (GA) <24 weeks.^12–16^ There is an urgent need for knowledge about ROP prevalence, severity, treatment and retreatment in these infants. The purpose of this study was to elucidate ROP, ROP treatment and risk factors for retreatments in infants born at GA <24 weeks.

## Materials and methods

### Study population and study procedures

The study group included 399 infants born at <24 weeks of GA between 2007 and 2018 who had fulfilled ROP screening in Sweden. In Sweden, ROP data are registered in the Swedish National Patient Registry for ROP (SWEDROP) through a standardised protocol by trained ophthalmologists who performed the screening examinations.^17^ Screening was performed according to national guidelines and consisted of dilated ocular fundus examinations.^18^ All infants were examined repeatedly until complete retinal vascularisation or until spontaneous or post-treatment regression of ROP. The revised International Classification of Retinopathy of Prematurity was used for classification, and the recommendations of the Early Treatment for Retinopathy of Prematurity Cooperative Group were followed for treatment.^19 20^ In this study, maximal ROP stage and time, method and centre for ROP treatment were retrieved from SWEDROP and validated in medical files. ROP treatment was performed at seven university hospitals with eye clinics and tertiary level Neonatal intensive care unit (NICU) units, hereafter referred to as ‘treatment centre’ (A-G). Birth weight (BW), GA, sex and neonatal morbidities were retrieved retrospectively from the medical files. Registered neonatal morbidities included intraventricular haemorrhage (IVH), necrotising enterocolitis (NEC), persistent ductus arteriosus (PDA) and bronchopulmonary dysplasia (BPD).

### Statistical analysis

Number and percentage are given for categorical variables, and for continuous variables, mean, median and range where applicable. For comparison between two groups, we used the Pearson’s χ^2^ test and Fisher’s exact test for dichotomous variables and Mann-Whitney U test for continuous variables. To determine trends of calendar year, linear regression models have been used for continuous variables and logistic regression for dichotomous variables, results are presented as related to a one-year increase. Univariate and multivariate logistic regression analyses were applied to evaluate the impact of different risk factors for ROP retreatment. Results were presented as ORs and 95% CI, and the level of signiﬁcance was set as a p value of <0.05. Pearson’s and Spearman correlation tests was used to estimate correlations between risk factors. The ﬁt of the models was checked with the Hosmer-Lemeshow goodness-of-ﬁt test. All analyses were carried out with IBM SPSS Statistics for Windows, V.25.0 (IBM Corp, Armonk, New York, USA).

Patients or the public were not involved in the design, or conduct, or reporting, or dissemination plans of our research.

## Results

Birth characteristics and ROP outcomes are provided in table 1. Among 399 infants, 188 (47.1%) were girls. Mean BW was 567 g (range 340–874 g), and mean GA was 23.2 weeks (range 21.9–23.9). Infants’ median number of ROP eye examinations was 14 (range 3–42).

**Table 1:** Birth characteristics and ROP outcomes in extremely preterm infants born 2007–2018 (n=399)

**Birth characteristics**	
Birth weight, mean (g)	567 (340 to 874)
Gestational age, mean (weeks)	23.2 (21.9 to 23.9)
Sex (female)	188/399 (47.1)
**ROP outcome**	
No ROP	34/399 (8.5)
ROP stages 1 and 2	125/399 (31.3)
ROP stage 3	227/399 (56.9)
ROP stages 4 and 5	13/399 (3.2)
ROP treatment	173/399 (43.4)
ROP retreatment	68/173 (39.3)

Values are presented as mean (max and min) or n (%).

ROP, retinopathy of prematurity.

### ROP outcome, treatment and retreatment

**Figure 1:**
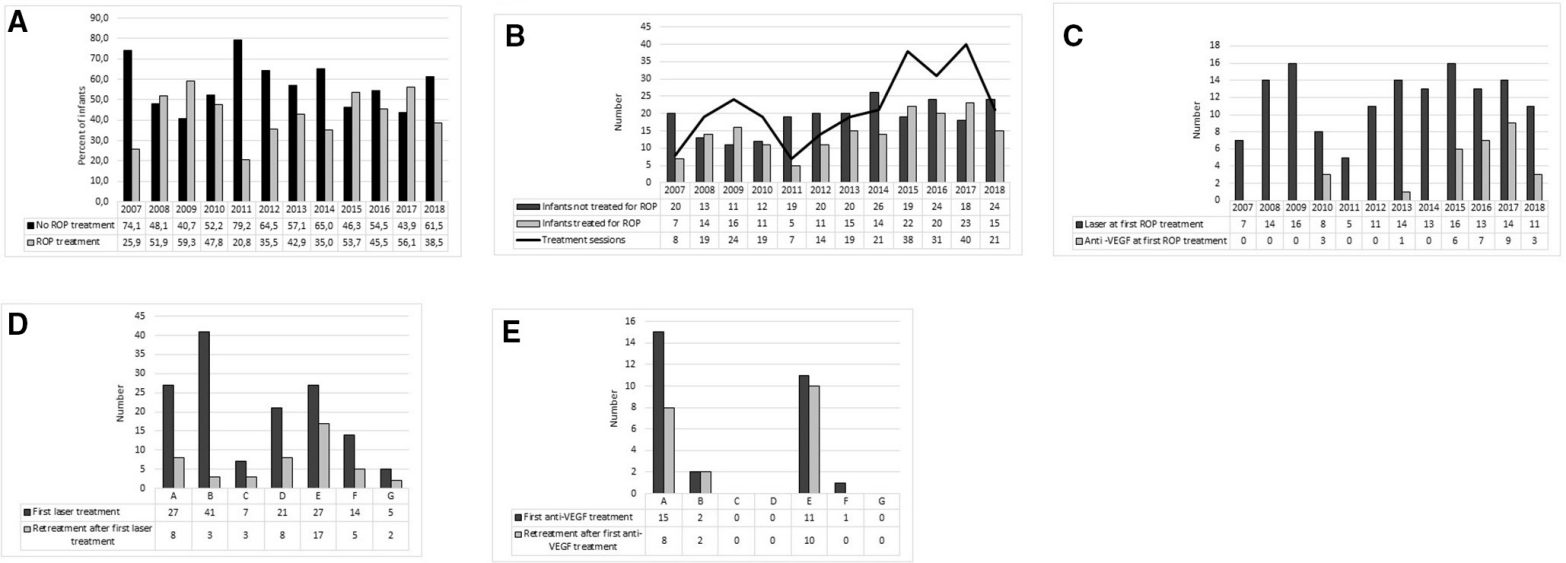
(A) Percent of infants receiving ROP treatments or not per infant’s birth year during 2007–2018 (n=399). (B) Number of infants (n=399) receiving ROP treatment or not and number of ROP treatment sessions during 2007–2018. (C) Number of infants receiving laser and anti-VEGF at first treatment during 2007–2018 (n=171). (D) Number of infants receiving laser as first treatment (n=142) and any retreatment during 2007–2018 at the seven treatment centres (A–G). (E) Number of infants receiving anti-VEGF as first treatment (n=29) and any retreatment during 2007–2018 at the seven treatment centres (A–G). anti-VEGF, anti-vascular endothelial growth factor; ROP, retinopathy of prematurity.

Altogether 365/399 (91.5%) developed some ROP stage and 173/399 (43.4%) received treatment for ROP. Retreatment was performed in 68/173 (39.3%) of the infants after first treatment. The incidence of ROP treatment did not change during the study period. However, the number of infants screened for ROP increased over the years (OR 1.82, 95% CI 1.07 to 2.56; p<0.001) as well as the number of infants treated (OR 1.83, 95% CI 0.27 to 3.39; p=0.026). We notised a trend in increased mean number of ROP treatments per infant over time however not significant (figure 1A, B).

### ROP treatment modality and retreatment rate

The first ROP treatment was performed at a median postnatal age (PNA) of 12.4 weeks (range 9.6–24.1 weeks) and a median postmenstrual age (PMA) of 35.7 weeks (range 32.0–47.7 weeks). PNA and PMA at first treatment and first retreatment related to treatment modality are presented in table 2. All infants were treated bilaterally. Seventeen infants had an especially complicated ROP course and were treated more than twice; 14 infants were treated three times and three infants were treated four times. The first treatment was laser therapy in 142/173 (82.0%) infants and anti-VEGF in 29/173 (16.8%) infants (p<0.001). Retreatment after the first treatment was more common in infants receiving anti-VEGF injections than laser (69.0% vs 32.4% (p<0.001)) (table 2). Retreatment after anti-VEGF occurred later, in median after 8.3 weeks compared with 2.2 weeks after laser (p=0.001). The longest duration before retreatment after anti-VEGF injection was 73.3 weeks, when peripheral vaso-proliferation was found with ophthalmoscopy during general anaesthesia for abdominal surgery. Online supplemental figure 1 presents a flow chart of ROP treatment and retreatments.

10.1136/bmjophth-2020-000695.supp1 Supplementary data

**Table 2:** Characteristics of first ROP treatment (n=173) and retreatment in association to postmenstrual and postnatal age

	Laser	Anti-VEGF	P value
Treatment method, n (%)	142/173 (82.0)	29/173 (16.7)	<0.001
PNA at treatment, median (weeks)	12.6 (14.9 to 24.0)	11.3 (9.6 to 24.1)	0.001
PMA at treatment, median (weeks)	36.0 (32.4 to 47.7)	34.1 (32.0 to 47.0)	<0.001
Retreatment rate, n (%)	46/142 (32.4)	20/29 (69.0)	<0.001
Time to retreatment, median (weeks)	2.2 (0.9 to 12.7)	8.3 (2.0 to 13.6)	0.001
PNA at retreatment, median (weeks)	15.1 (11.1 to 27.6)	19.6 (13.3 to 26.9)*	0.001
PMA at retreatment, median (weeks)	38.6 (34.3 to 51.1)	42.4 (36.1 to 49.3)*	0.001

Values are presented as median (max and min) or n (%).

*One infant excluded receiving retreatment at PNA 86.7 weeks and PMA 109.6 weeks.

anti-VEGF, antivascular endothelial growth factor; PMA, postmenstrual age; PNA, postnatal age.

Of the 29 infants receiving anti-VEGF as first treatment, 4/29 (13.8%) infants received bevacizumab (0.4–0.625 mg) and 25/29 (86.2%) infants received ranibizumab (0.2–0.5 mg). First anti-VEGF treatments (n=3) were performed in 2010 (all with bevacizumab). Figure 1C presents number of laser and anti-VEGF injections as first treatment by the infant’s birth year.

### Aggressive posterior retinopathy of prematurity

Twenty-two infants (5.5%) developed APROP. Of these as first treatment, 16/22 (72.7%) received anti-VEGF, 5/22 (22.7%) received laser and one infant received both laser and anti-VEGF. Six infants diagnosed with APROP 6/22 (27.3%) progressed to retinal detachment, constituting 46.2% (6/13) of infants with retinal detachment. Three of these six infants had been primarily treated with laser, two with anti-VEGF injection and one infant with both laser and anti-VEGF injection. Two infants with APROP developed retinal detachment and later endophthalmitis in one eye each, after anti-VEGF injection, progressing to phthisis (online supplemental figure 2).

10.1136/bmjophth-2020-000695.supp2 Supplementary data

### Surgical interventions due to retinal detachment

Twenty eyes in 13 infants (3.2%) progressed to retinal detachment (unilateral in six infants and bilateral in seven infants). Three infants were subjected to surgical interventions. One infant with bilateral stage 4B underwent vitrectomy in one eye and did not become blind in either eye. Two infants had bilateral stage 5 ROP, and one of them underwent bilateral vitrectomy and lensectomy. The other infant with stage 5 had total retinal detachments after two sessions of laser therapy and underwent scleral buckling in one eye. Both these infants were blind in both eyes. In online supplemental figure 2, details about infants with a retinal unfavourable outcome are presented, and in online supplemental table 1, details about infants with retinal detachment are presented.

10.1136/bmjophth-2020-000695.supp3 Supplementary data

### Centre and treatment modality

Choice of treatment method differed among treatment centres. The use of anti-VEGF injections as first treatment varied from none to 35.7%. Treatment numbers and modalities regarding treatment centres are presented in figure 1D–E. There was a significant centre difference in the prevalence of APROP (p=0.022) but not in GA, BW or sex of infants treated at different centres. Retreatment rate was influenced by treatment centre only when laser was the initial treatment modality. The rate of retreatment after laser therapy ranged from 7.1% to 63.0% depending on the treatment centre, figure 1D. The rate of retreatment after first anti-VEGF injection was overall 69.0% (figure 1E).

### Risk factors for retreatment

In univariate logistic regression analysis, none of the following variables were significant risk factors for retreatment: GA, BW, sex, IVH, BPD, NEC or PDA. However, APROP (OR 4.003, 95% CI 1.537 to 10.424, p=0.005), anti-VEGF treatment (OR 4.686, 95% CI 1.980 to 11.089, p<0.001) and treatment centre (OR 1.065, 95% CI 1.026 to 1.105, p=0.001) were identified as risk factors. In multivariate analysis, treatment centre persisted as the major risk factor for retreatment overall (OR 1.087, 95% CI 1.043 to 1.134, p<0.001). Treatment centre remained as a risk factor after first laser treatment (OR 1.085, 95% CI 1.037˗1.135, p<0.001) but not after anti-VEGF as first treatment (data not shown). For details, see table 3.

**Table 3:** Univariate and multivariate logistic regression analysis of factors that might influence ROP retreatment

Variable	Univariate analysis		Multivariate analysis		Multivariate analysis laser treatment as first treatment	
	P value	OR (95% CI)	P value	OR (95% CI)	P value	OR (95% CI)
Gestational age (weeks)	0.635	0.858 (0.455 to 1.616)	–	–	–	–
Birth weight (50 g increment)	0.062	0.815 (0.658 to 1.010)	0.200	0.852 (0.667 to 1.089)	0.113	0.808 (0.621 to 1.052)
Sex	0.838	0.938 (0.509 to 1.729	–	–	–	–
APROP	0.005	4.003 (1.537 to 10.424)	0.119	2.733 (0.771 to 9.688)	0.269	2.901 (0.439 to 19.167)
Treatment method (anti-VEGF)	<0.001	4.686 (1.980 to 11.089)	0.057	3.086 (0.967 to 9.852)	–	–
Treatment centre	0.001	1.065 (1.026 to 1.105)	<0.001	1.087 (1.043 to 1.134)	<0.001	1.085 (1.037 to 1.135)
IVH	0.468	1.259 (0.676 to 2.346)	–	–	–	–
BPD	0.806	1.173 (0.330 to 4.173)	–	–	–	–
NEC	0.684	1.161 (0.564 to 2.391)	–	–	–	–
PDA	0.321	0.622 (0.244 to 1.589)	–	–	–	–

Data presented with p values and ORs and 95% CI.

Covariates with p<0.2 in univariate analysis were entered in a multivariate logistic analysis.

anti-VEGF, antivascular endothelial growth factor; APROP, aggressive posterior retinopathy of prematurity; BPD, bronchopulmonary dysplasia; IVH, intraventricular haemorrhage; NEC, necrotising enterocolitis; PDA, patent ductus arteriosus; ROP, retinopathy of prematurity.

## Discussion

In this national study comprising preterm infants born at GA <24 weeks, the rate of ROP, ROP treatment, retreatment and unfavourable retinal outcomes were substantial. Of 399 infants born at GA <24 weeks, 91.5% developed any stage ROP and 65.8% of those developed severe ROP (stage 3 or more). Altogether 43.3% and 72.2% of those with severe ROP underwent ROP treatment. In 20 eyes of 13 infants (3.2%) ROP progressed to retinal detachment despite repeated treatment attempts. APROP was diagnosed in 22 (5.5%) infants and progressed to retinal detachment in six infants. Two infants developed unilateral endophthalmitis and phthisis after anti-VEGF injections.

Previous studies on ROP have included a limited number of infants born at GA <24 weeks. Due to different policies regarding active perinatal care and ROP treatment at these low GAs over time and between hospitals and countries, comparisons with other cohorts of extremely preterm infants must be made with caution. In a previous Swedish population-based study during 2004–2007, 53/58 (91.4%) infants with GA <24 weeks at birth developed some stage of ROP and 27/58 (46.5%) underwent treatment.^6^ Ishii *et al* reported that of Japanese infants born at GA <24 weeks in 2003–2005 (n=320), 27.5% required ROP treatment, while Miller *et al* reported in a much smaller group of patients (n=23) that the cumulative probability of receiving laser therapy was nearly 46.0% if born at GA <24 weeks in the USA, 2006–2008.^13 15^ In a study from England in 2006 (the EPICure study), Costeloe *et al*^12^ reported that 31.9% of infants born at GA <24 weeks received laser treatment for ROP (n=69). In the current study from Sweden from 2007 to 2018, 43.3% of the most immature infants were treated. The proportion of screened infants who were treated yearly varied during the study period, ranging from 20.8% of infants born in 2011 to 59.3% of infants born in 2009. We found no increasing in incidence of ROP treatment during the study period; however, the number of infants screened increased over time, which indicate increased survival rates but not decreased morbidity rates. We suspect that the impact of the new routines with increased oxygen saturation target levels from 88%–92% to 91%–95% that were implemented in most parts of Sweden during 2014 have probably affected treatment need. Holmström *et al* reported a nearly doubled increase in incidence of treatment for ROP in 2015 in one Swedish healthcare region, as compared to the years before the new oxygen routines (2008–2013), while the incidence remained stable in one region which did not implement the new oxygen routines.^21^

Survival rates may be influenced by many factors such as an obstetrician’s willingness to intervene to rescue the fetus and the neonatologist’s policy regarding initiation of neonatal intensive care as well as the quality of neonatal care for very immature infants.^1 2^ All these variables influence the health of the surviving infants. We found an increased number of infants screened and treated for ROP, and a trend of higher rates of retreatment in the later years of the study period that may be due to the increase in anti-VEGF treatment, which is known to have a high risk of recurrence. Thus, there has been an increasing workload for ophthalmologists performing ROP screening and treatment in Sweden in recent years. This is worrying, since there is a general shortage of ophthalmologists willing to perform ROP screening in this and many other countries.

In Sweden, the first intravitreal injection of anti-VEGF for ROP was performed in 2010. In 2015, anti-VEGF treatment became more frequently used as first choice of treatment for APROP and central ROP. In our cohort, the retreatment rate was 32.4% after laser and 69.0% after anti-VEGF, which to our knowledge has previously not been reported exclusively in infants with GA <24 weeks. Lower recurrence rates have been reported in cohorts comprising infants born with a wider range of GA.^22^ It is well known that recurrence is more common after anti-VEGF injections than after laser treatment.^23–25^ In the Rainbow trial, which formed the basis for approving the anti-VEGF drug ranibizumab for ROP, treatment recurrence rates were 18.9% after laser treatment and 31.1% after ranibizumab treatment.^11^ The high recurrence rate after anti-VEGF in our study may be partly due to the high percentage (5.5%) of infants with APROP, the most aggressive form of ROP with high risk for recurrence after treatment.^26 27^ In the current study, the median time to retreatment after first anti-VEGF injection was 8.3 weeks compared with 2.2 weeks after laser therapy. In the Rainbow trial, the time to recurrence after the first anti-VEGF treatment varied between 4.1 and 18.2 weeks (median 8.0 weeks).^11^ In our cohort, one infant was found to have peripheral vasoproliferation 73.3 weeks after ant-VEGF injection. Long-term follow-up is a logistic clinical problem, and there are no general guidelines for how long infants need to be followed after anti-VEGF injections.

Rate of recurrence after the first treatment varied among the seven centres where treatment was performed. Only two centres used anti-VEGF as first treatment in more than two patients and the proportion of retreated patients at those centres were 8/15 (53.3%) and 10/11 (90.9%), respectively. The retreatment rate after the first laser treatment varied from 7.1% to 63.0% among centres. In a recent national study, it was found that in 11/17 preterm infants who had become visually impaired, the ROP screening and/or treatment process had been suboptimal.^7^ In fact, in 10 of 17 visually impaired infants, the first laser treatment was considered suboptimal or untimely. These results are in line with the newly published study by Spandau *et al*,^28^ who demonstrated that treatment failure of type 1 ROP was due to inadequate laser treatment in 8/10 cases, for example, undertreatment, overtreatment or skip lesions. Hence, correct laser treatment is crucial and might be facilitated by using wide-angle photography after treatment, before finalising the procedure to identify areas of undertreatment or skip lesions (Gränse *et al*, in manuscript). All aspects of optimal screening including the ophthalmologist’s judgement of ROP staging, the presence of plus disease, APROP and need for treatment are essential to ensure timely treatment and ensuring the best possible outcome.^29 30^

In our cohort, 20 eyes of 13 infants had unfavourable retinal outcomes. These thirteen infants developed retinal detachment in one or both eyes, and two of those infants also developed endophthalmitis after anti-VEGF injection in one eye each. We reviewed these infants’ medical records and confirmed, in a majority of cases, the presence of treatment failures and/or incomplete follow-up in accordance with the findings reported by Norman *et al*.^7^

In this vulnerable group of infants, infants were subjected to a median of 14.0 ROP examinations (range 3–42). During the study period, a total of 6061 ROP examinations were performed. Altogether, 261 ROP treatments were performed in 173 infants. The ROP examinations are known to be stressful and painful for the infant. During and after ROP examination, fluctuant blood pressure, increased pulse rate, desaturation and increased need for oxygen supplementation occur.^31 32^ Laser therapy is regularly performed under general anaesthesia in Sweden, and most centres use general anaesthesia also for anti-VEGF injections. Infants undergoing laser treatment have been found to be at high risk of intraoperative and postoperative adverse events like hypotension, bradycardia and apnoea.^33^ Repeated anaesthetic procedures may affect the infants’ neurodevelopment, and there are concerns about the long-term effects of anti-VEGF treatment on neurodevelopmental outcome.^34^ Thus, it is crucial that the ROP screening and treatment procedures are as efficient and gentle as possible, especially as several authors have stressed that ROP is a biomarker for brain volumes at term and later neurodevelopmental outcomes.^35 36^

### Strengths and limitations

The study has a retrospective design, which is a limitation. The strengths are: prospectively collected validated data with national coverage for more than 10 years, follow-up of infants when moved between hospitals, patient register structured protocols (SWEDROP) and file review of all neonatal diagnoses.

## Conclusions

The increasing number of very immature infants with high incidence of sight-threatening ROP warranting treatment is worrying. The variation in retreatment rate between centers indicate that laser treatment timing and technique may be improved in some settings with possible positive effects on outcome. In addition, as severe ROP is not just a blinding eye disease but also a marker of impaired central nervous system development, the findings of the present study suggest that thorough neurodevelopmental investigation is warranted in this population.

## Data Availability

All data relevant to the study are included in the article or uploaded as supplementary information.
