# Supplementary figures and images for: High rate and large intercentre variability in retreatment of retinopathy of prematurity in infants born <24 gestational weeks
Source: BMJ Open Ophthalmol. 2021 Apr 21;6(1):e000695. doi: 10.1136/bmjophth-2020-000695 (PMC8070879; doi:10.1136/bmjophth-2020-000695)

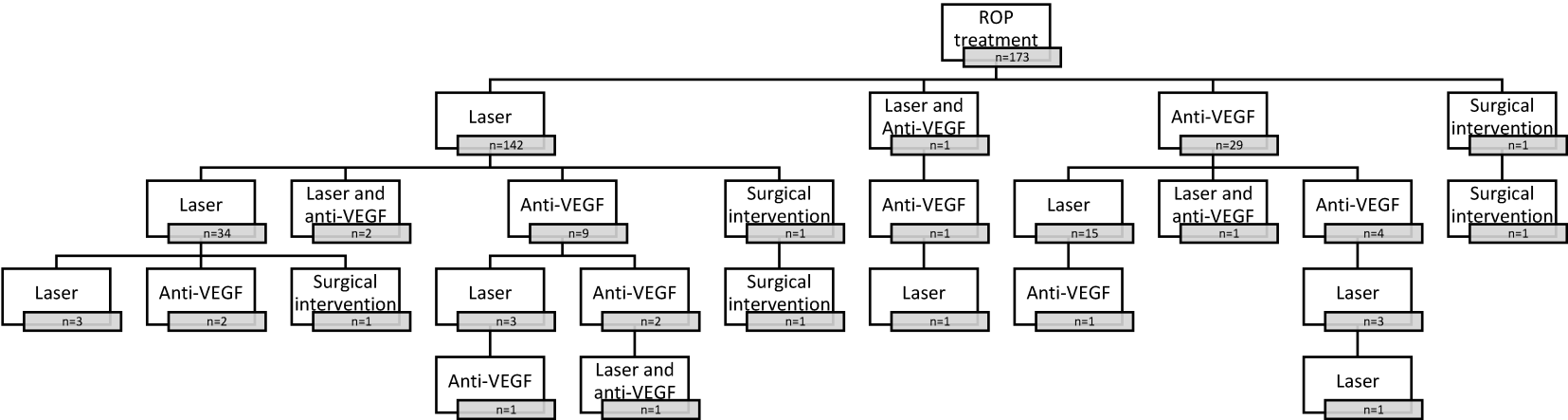

Supplement: Supplementary data [file bmjophth-2020-000695supp001.pdf]

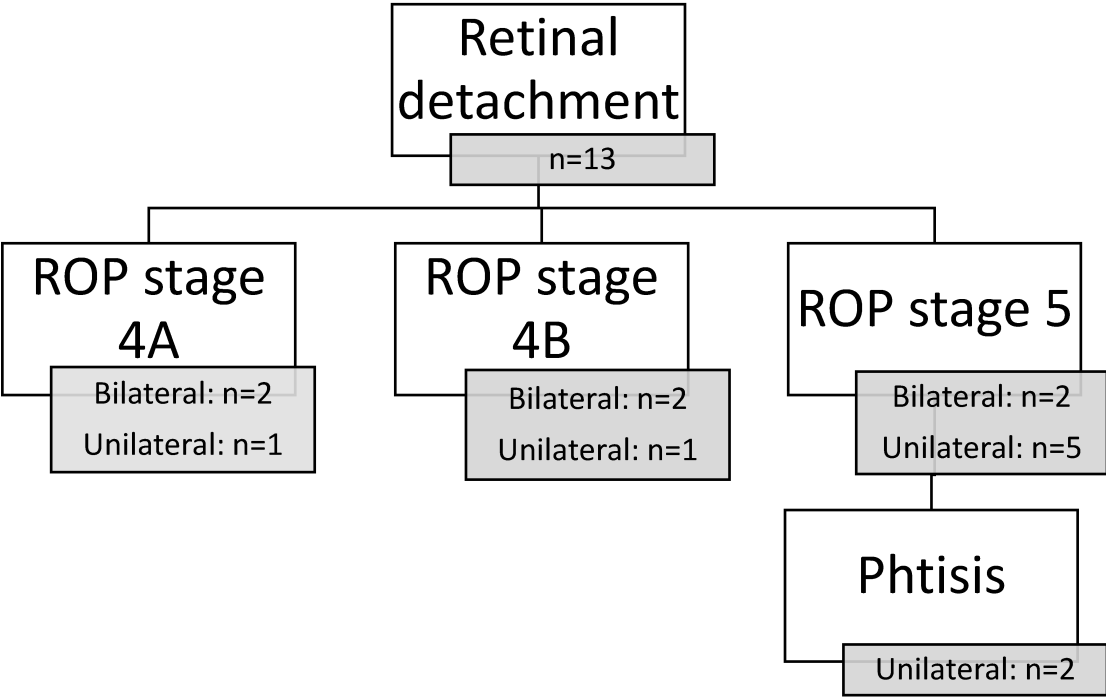

Supplement: Supplementary data [file bmjophth-2020-000695supp002.pdf]
