# Supplementary material for: High rate and large intercentre variability in retreatment of retinopathy of prematurity in infants born <24 gestational weeks
Source: BMJ Open Ophthalmol. 2021 Apr 21;6(1):e000695. doi: 10.1136/bmjophth-2020-000695 (PMC8070879; doi:10.1136/bmjophth-2020-000695)
Supplement: Supplementary data [file bmjophth-2020-000695supp003.pdf]

Supplemental table 1: Characteristics and treatment modality in infants developing retinal detachment due to retinopathy of prematurity (ROP)

Sex	Birth weight, grams	Gestational age, weeks	Presence of APROP	Maximum stage ROP right eye	Maximum stage ROP left eye	No of ROP treatments	Treatment no 1	Treatment no 2	Treatment no 3	Treatment no 4	Additional information
Girl	533	22.71		Stage-4A	Stage-4A	3	Laser	Vitrectomy right eye	Vitrectomy and lensectomy right eye		
Girl	565	23.43		Stage-4A	Stage-3	1	Laser				
Boy	585	23.43		Stage-5	Stage-3	2	Laser	Laser			
Girl	490	22.14		Stage-4B	Stage-4B	2	Laser	Laser			
Boy	685	23.14		Stage-5	Stage-5	2	Vitrectomy and lensectomy left eye	Vitrectomy and Lensectomy right eye			
Boy	675	23.43	APROP	Stage-4B	Stage-3	3	Laser	Laser	Anti-VEGF		
Girl	512	23.86	APROP	Stage-5	Stage-3	2	Anti-VEGF	Laser			Endophthalmitis and phthisis right eye
Girl	560	23.29		Stage-5	Stage-5	3	Laser	Laser	Scleral buckling right eye		
Boy	565	22.43	APROP	Stage-4A	Stage-5	2	Anti-VEGF	Laser			
Girl	626	23.29	APROP	Stage-3	Stage-4A	1	Laser				
Girl	566	23.29	APROP	Stage-3	Stage-5	3	Laser	Anti-VEGF	Anti-VEGF		Endophthalmitis and phthisis left eye
Girl	595	23.57		Stage-4A	Stage-5	4	Laser	Anti-VEGF	Anti-VEGF	Laser and anti-VEGF	
Boy	507	23.29	APROP	Stage-4B	Stage-4B	3	Laser and anti-VEGF	Anti-VEGF	Laser		

Abbreviations: APROP, aggressive posterior retinopathy of prematurity; no, number; ROP, retinopathy of prematurity; VEGF, vascular endothelial growth factor
